# Effects of Microalgae Addition and Fish Feed Supplementation in the Integrated Rearing of Pacific White Shrimp and Nile Tilapia Using Biofloc Technology

**DOI:** 10.3390/ani12121527

**Published:** 2022-06-13

**Authors:** Vitor F. Silva, Patriula K. M. Pereira, Mateus A. Martins, Marco A. d. Lorenzo, Herculano Cella, Rafael G. Lopes, Roberto B. Derner, Paola Magallón-Servín, Felipe d. N. Vieira

**Affiliations:** 1 Marine Shrimp Laboratory, Federal University of Santa Catarina (UFSC), Florianópolis 88061-600, Brazil; vfernandessilva95@gmail.com (V.F.S.); patriulakaliana@hotmail.com (P.K.M.P.); m.aranha.martins@gmail.com (M.A.M.); m.a.lorenzo@ufsc.br (M.A.d.L.); 2 Laboratory of Algae Cultivation, Federal University of Santa Catarina (UFSC), Florianópolis 88061-600, Brazil; hercu.ufsc@yahoo.com.br (H.C.); rafael.lopes@ufsc.br (R.G.L.); roberto.derner@ufsc.br (R.B.D.); 3 CONACYT-Centro de Investigaciones Biológicas del Noroeste, La Paz 23205, Mexico; pmagallon@cibnor.mx

**Keywords:** *Litopenaeus vannamei*, *Oreochromis niloticus*, marine shrimp, BFT

## Abstract

**Simple Summary:**

In biofloc technology systems, organic matter and inorganic substances accumulate in the culture unit mainly due to low water exchange, feed input, high stocking densities, and the level of organic carbon that subsequently increase the bacterial biomass. The organic and inorganic matter in suspension are maintained in a limited concentration to avoid negative effects on animal performance and water quality. This requires the occasional removal of excess solids, which produces an effluent enriched in nutrients, such as nitrogen and phosphorus. The use of integrated aquaculture in which the residues of one species are used as a source of nutrients for another species is an approach which aims to minimize this production of waste. Still, there are aspects of integrated cultures that have to be studied, such as the provision of feed for the different species. Therefore, we evaluated the addition or not of fish feed and microalgae in an integrated shrimp and fish culture regarding animal growth performance and water quality. We found that biofloc is nutritive for fish, but feed is necessary to improve its growth. Moreover, microalgae improved fish survival. However, both of factors did not affect the solids production.

**Abstract:**

This study aims to evaluate a Pacific white shrimp and Nile tilapia integrated system using biofloc technology with or without the addition of the microalgae *Scenedesmus obliquus* and with or without fish feed supplementation in a two-factor 62-day experiment. The shrimp (2.16 ± 0.01 g) were reared under a density of 400 shrimp m^−3^ and the fish (1.53 ± 0.12 g) were reared under a density of 522 fish m^−3^. The microalgae was added to the culture water two times a week. Growth performance, sludge production, and water microbiology were evaluated. Fish feed and the microalgae addition improved fish final biomass in 58% and 14%, respectively (*p* < 0.05). Fish survival was significantly higher when microalgae was added (93.9 ± 1.8%) compared with the treatments without microalgae addition (86.2 ± 7.6%) (*p* < 0.05). The yield of the overall system was higher in the treatments with fish feed supplementation (4.2 ± 0.2 kg m^−3^) compared with no addition (3.9 ± 0.2 kg m^−3^) (*p* < 0.05). These results suggest that fish feed supplementation at the rate of 1% of the biomass and microalgae inoculation can improve fish growth performance and system yield, without affecting sludge production and water microbiology. This work is an expansion of a conference paper with the same title.

## 1. Introduction

Aquaculture using conventional production systems—e.g., semi-intensive or intensive systems using earthen ponds and water exchange as the main water quality control strategy—has the potential to contribute to environmental degradation, due to high water exchange rates, nutrient enriched effluents, and the indiscriminate use of pharmaceutical substances, which can cause pollution and eutrophication of natural environments [1,2,3]. In this context, modern aquaculture has the responsibility of increasing production through sustainable approaches as a result of the worldwide increase in fish and shellfish consumption and the need of maintaining fisheries stocks in a more sustainable level.

Intensification along with minimum water exchange systems, new feeding strategies that combine natural food supplementation and restriction of commercial manufactured feeds, and the integration of different species are strategies that can potentially increase aquaculture production in a sustainable manner [3,4]. In this regard, the use of biofloc technology (BFT) has increased in recent years [5,6]. Some of its main benefits are increased biosecurity and yield through a more efficient use of water and land as a result of low water exchange rates and high stocking densities [2].

In the BFT systems, organic matter and inorganic substances accumulate in the culture unit mainly due to the input of feed, stocking density, and the level of organic carbon (C) that subsequently impact the bacterial biomass. The flocs in suspension are measured using total suspended solids (TSS). High TSS concentrations can negatively affect animal performance and water quality [7,8]. This requires the occasional removal of excess solids, which produces an effluent enriched in nutrients, such as nitrogen and phosphorus [9]. Thus, strategies must be adopted to reuse these wasted nutrients within the system in order to reduce environmental impacts and increase its sustainability.

The integrated culture of different species aims to optimize the use of nutrients generated by a primary fed species by employing other species that can take advantage in the excess nutrients, thus improving the use of resources of the overall production system [3,10,11]. Two species with the potential to be cultured in integration are Pacific white shrimp (*Litopenaeus vannamei*) and Nile tilapia (*Oreochromis niloticus*) [12]. Poli et al. [13] evaluated different Nile tilapia densities (10, 20, 30% of the biomass relative to the shrimp biomass) and found gains in the overall system yield and nutrient (i.e., nitrogen and phosphorus) recovery, along with a reduction in the sludge: biomass relationship as the fish density increased. Martins et al. [14] reported differences in the overall yield, water microbiology and recovery of nutrients between heterotrophic and mature biofloc systems integrating the same species, in which the heterotrophic system improved tilapia growth performance, the overall recovery of nutrients and modulated the water bacterial community. In both studies, the fish were fed at the rate of 1% of their biomass and the feed conversion ratio (FCR) observed by the authors ranged between 0.15 and 0.38, indicating a relevant contribution of bioflocs in nutrition of the fish. However, there are no studies assessing if the fish can be reared without feed supplementation and still maintain adequate performances without, consequentially, negatively impacting the overall integrated system, which would allow a reduction in the system inputs.

Tilapia are known for their diverse feeding behavior, with studies showing that phytoplankton and suspended organic matter are important components of their diets in the natural environment [15,16], with food selection by fish depending on factors such as size and palatability [17]. Numerous studies have shown that tilapia can be reared using biofloc technology and is capable of taking advantage of the microbial aggregates as a food source [1,13,14].

The microalgae *Scenedesmus obliquus* is a freshwater green algae rich in protein and lipid contents, in addition to the carotenoid lutein [18]. Works employing the microalgae *S. obliquus* in the culture of Nile tilapia exhibited promising results. For instance, the partial substitution of fish meal for *S. obliquus* meal (50% substitution) improved Nile tilapia growth performance [17]. Its inoculation along with *Chlorella* sp. in a biofloc system benefitted the immune system of the same fish species [19]. Moreover, its application in consortium with bacteria and other microalgae for the biological treatment of effluents has also demonstrated a reduction in nutrient concentration, such as nitrogen, phosphorus, and carbon [20]. The aforementioned studies indicate that the addition of microalgae species to the water of biofloc-based integrated systems may enhance biofloc attractiveness and nutritional composition, increasing natural food consumption by fish and improving their growth performance.

Furthermore, the bacterial community of biofloc systems includes genera of potentially pathogenic species for fish and shrimp. The bacteria of the genus *Vibrio* are considered to be opportunists, particularly in marine and estuarine environments, expressing their virulence in stressed animals, such as can occur under unstable environments and high stocking densities, causing negative economic impacts as a result of massive mortalities, necrosis, and growth impairment [21,22]. Some authors suggest that the presence of tilapia in the culture water reduces the incidence of *Vibrio* spp. due to antibacterial properties of the fish mucus and antagonistic effects of fish gut microbiome metabolites [23,24,25].

Therefore, this study aimed to evaluate the effects of Nile tilapia feed supplementation and *S. obliquus* inoculation in the integrated culture of *O. niloticus* and *L. vannamei* using biofloc technology, as regards growth performance, sludge production, and water microbiology.

## 2. Materials and Methods

The experiment was conducted at the Marine Shrimp Laboratory (LCM) (27°34′56.0″ S 48°26′29.7″ W), part of the Aquaculture Department of the Federal University of Santa Catarina (UFSC), Brazil.

### 2.1. Biological Material

#### 2.1.1. Shrimp

*Litopenaeus vannamei* nauplii (Speedline Aqua lineage), were acquired from the company Aquatec Ltd.a. (Rio Grande do Norte, Brazil). The animals were reared in a biofloc system until reaching a mean weight of 2 g.

#### 2.1.2. Fish

The Nile tilapia (*Oreochromis niloticus*) juveniles (GIFT lineage) were acquired from the company Acqua Sul Piscicultura (Ilhota, Brazil), with a mean weight of 1.53 ± 0.12 g. At the laboratory, they were acclimatized to a salinity of 15 g L^−1^ under a rate of 2 g L^−1^ day^−1^ [26] through the addition of sea water (34 g L^−1^).

#### 2.1.3. Microalgae

The *Scenedesmus obliquus* microalgae were obtained from the strain bank of the Algae Cultivation Laboratory (LCA), part of the Federal University of Santa Catarina (UFSC), located at Barra da Lagoa, Florianópolis, Santa Catarina, Brazil (27°34′56.1″ S 48°26′29.2″ W).

Microalgae were cultivated in thin layer cascade system (TLC) using a fed-batch method [27] with the LCA-AD culture medium (4 N; P/3), adapted from the original BBM medium [28]. The final concentration of the culture medium was the following: NaNO_3_ 1000 mg L^−1^; CaCl_2_.2H_2_O 25 mg L^−1^; MgSO_4_.7H_2_O 75 mg L^−1^; K_2_HPO_4_ 25 mg L^−1^; KH_2_PO_4_ 58.3 mg L^−1^; NaCl 25 mg L^−1^; Na_2_EDTA.2H_2_O 50 mg L^−1^; KOH 31 mg L^−1^; FeSO_4_.7H_2_O 4.98 mg L^−1^; ZnSO_4_.7H_2_O 0.00882 mg L^−1^; MnCl_2_.4H_2_O 0.00144 mg L^−1^; (NH_4_)_6_MoO_7_O_24_.H_2_O 0.00661 mg L^−1^; CuSO_4_.5H_2_O 0.00157 mg L^−1^; and Co(NO_3_)_2_.6H_2_O 0.0004 mg L^−1^. Nutrients were replaced daily based on nitrate consumption, whose concentration was determined from samples of the filtered medium, according to the colorimetric method (HACH^®^) with the PERMACHEM^®^ Reagent NitraVer (Loveland, CO, USA) through absorbance readings at 410 nm and the proper standard curve for each nutrient.

Acclimation of the microalgae was performed before inoculation to the integrated system. Two days before every addition, 40 L of the microalgae culture was transferred to two 20 L bottles with constant aeration, after which seawater (34 g L^−1^) was added to increase salinity at the rate of 5 g L^−1^ day^−1^. At the day of inoculation, the culture salinity was 10 g L^−1^. The integrity of the microalgae cells was observed using an optical microscope before the transfer to the experimental units. Aiming at a final concentration of 5 mg L^−1^ at the experimental units, the volume of microalgae culture to be added was calculated based on measuring the culture biomass in the 20 L bottles (estimated through turbidity) and based on the following formula (1):

V_c_ = (B_eu_ × V_eu_) ÷ B_c_, (1)

where,

V_c_ = microalgae culture volume to be added to the experimental units (L);

B_eu_ = desired final microalgae biomass at the experimental units (5 mg L^−1^);

V_eu_ = water volume of the experimental units (890 L);

B_c_ = Microalgae culture biomass in the 20 L bottles after acclimation (mg L^−1^).

### 2.2. Experimental Design

A 62-day factorial experiment was conducted, in which two factors, each with two levels, were evaluated in quadruplicate: the addition of the microalgae *Scenedesmus obliquus* to the tank water and the supplementation of fish feed, totaling four treatments, namely: (1) No addition of microalgae and no addition of fish feed; (2) no addition of microalgae and addition of fish feed; (3) addition of microalgae and no addition of fish feed; and (4) addition of microalgae and addition of fish feed. No alteration was made in the feeding of shrimp in any of the treatments.

The microalgae addition was performed twice a week with the aim of maintaining a stable concentration throughout the experiment. The final *S. obliquus* concentration of 5 mg L^−1^ (dry biomass) at the experimental units was chosen so as to not affect the production of solids.

The shrimp (2.16 ± 0.01 g) were stocked under a density of 400 shrimp m^−3^ (320 shrimp tank^−1^) and the fish (1.53 ± 0.12 g) were stocked under a density of 522 fish m^−3^ (47 fish tank^−1^).

In the treatments in which the fish received feed, they were fed once a day at the rate of 1% of their biomass, using a commercial feed (Guabitech Inicial 1 mm, 45% crude protein). Shrimp in all treatments were fed four times a day according to a feeding table [29] with a 40% crude protein commercial feed (Guabitech Inicial J40) until they reached a mean weight of 3 g, after which the feed was substituted with a 35% crude protein one (Guabi Poti Guaçu 1.6 mm).

**Figure 1:**
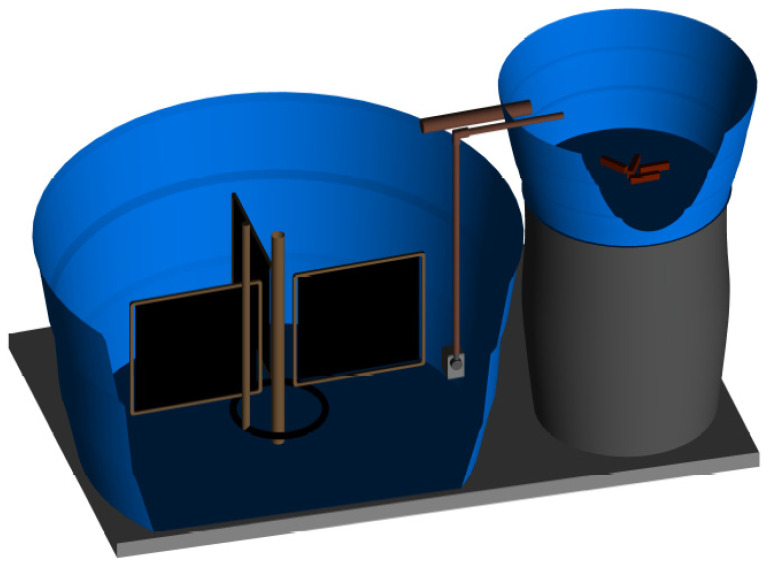
Schematic drawing of the experimental units used in a Pacific white shrimp (*Litopenaeus vannamei*) and Nile tilapia (*Oreochromis niloticus*) integrated culture using biofloc technology for 62 days, in which the addition or no addition of both microalgae and fish feed were evaluated in a factorial design.

The experimental units (Figure 1) were composed of 1000 L tanks (800 L of useful volume) and 100 L tanks (90 L of useful volume) for the shrimp and fish, respectively, located in an agricultural greenhouse and covered with shade cloth. A submerged pump (Sarlo-Better 650 L h^−1^) placed in the shrimp tanks pumped the water to the fish tanks, which returned to the former by gravity, maintaining a 24 h day^−1^ recirculation. The shrimp units were equipped with artificial substrates made of high-density polyester (Needlona^®^), comprising 80% of the tank surface area. A circular microperforated hose in the shrimp unit and four air-stones in the fish unit connected to a central blower maintained dissolved oxygen concentrations above 5 mg L^−1^ and the bioflocs in suspension. Water temperature was kept at 29.2 ± 1.2 °C through an 800 W heater in the shrimp units.

Before the experiment began, the shrimp tanks were filled with approximately 40% of water from a matrix biofloc tank. Then, shrimp were stocked and acclimatized to a salinity of 15 g L^−1^ with freshwater according to the procedure of Van Wyk [30].

Similarly, the fish tanks were filled with 40% of water from the biofloc matrix tank. The remaining was filled with freshwater before the animals were stocked.

The physico-chemical characteristics of the biofloc matrix tank water were: total ammonia-N 0.37 mg L^−1^; nitrite (N-NO_2_^−^) 0.34 mg L^−1^; nitrate (N-NO_3_^−^) 11.15 mg L^−1^; TSS 465 mg L^−1^; pH 8.00; alkalinity 160 mg L^−1^ and salinity 35.2 g L^−1^. Freshwater used in the acclimation was provided by the water works company of Florianópolis, SC, Brazil. Salinity was controlled through the addition of freshwater to compensate water lost through evaporation, while alkalinity was kept above 120 mg L^−1^ by the addition of calcium hydroxide.

### 2.3. Chlorophyll-a Analysis

One hundred mL water samples collected from the experimental units were allowed to settle for 15 min. Subsequentially, a 10 mL aliquot from each unit was collected and filtered with a glass fiber microfilter GF-1 (Macherey-Nagel). The photosynthetic pigment chlorophyll-*a* was extracted with acetone (90%) (Quimex, Mogi das Cruzes, SP, Brazil) and quantified in a photocolorimeter (Alfakit, Florianópolis, SC, Brazil), using the 664 and 630 nm wavelengths [30]. The chlorophyll-*a* concentration was calculated according to the methodology of Jeffrey and Humphrey [31].

### 2.4. Water Quality Analysis

Dissolved oxygen and temperature were measured twice a day with a digital oximeter (YSI Pro20). Alkalinity and pH were measured twice a week using a titration method [32] and a digital pHmeter (Tecnal, Piracicaba, SP, Brazil). Total ammonia and nitrite were measured twice a week through the indophenol method [33] and Griess reaction [34], respectively. Total, volatile, and fixed suspended solids were measured twice a week using the gravimetric method [32]. Salinity was measured once a week with a refractometer. Nitrate and orthophosphate were measured at the beginning, middle, and end of the experiment with the kit Hach NitraVer 5 (nitrate reagent powder pillows, Loveland, CO, USA) and the ascorbic acid method [32], respectively.

### 2.5. Growth Performance

Shrimp, fish, and the overall integrated system performances were evaluated at the end of the experiment to estimate final mean weight, weekly growth rate, daily growth coefficient (DGC), biomass, yield, FCR, and survival. Shrimp and fish were weighed weekly and fortnightly, respectively, to assess their growth and adjust the feeding ratios. The formulae used to assess the performances were:

Final mean weight^s,f^ (g) = (biomass) ÷ (number of animals), (2)

Weekly growth rate^s^ (g week^−1^) = (weight gain) ÷ (weeks of rearing), (3)

Daily growth coefficient^s,f^ (% day^−1^) = [(final weight^1/3^ − initial weight^1/3^) ÷ days of rearing] × 100, (4)

Biomass^s,f^ (g) = final mean weight × final number of animals, (5)

Biomass^is^ (g) = (final fish biomass + final shrimp biomass), (6)

Yield^s,f^ (kg m^−3^) = (final biomass) ÷ (tank volume), (7)

FCR^s,f^ = (feed provided) ÷ (final biomass), (8)

Yield^is^ (kg m^−3^) = (final fish biomass + final shrimp biomass) ÷ (fish tank volume + shrimp tank volume), (9)

FCR^is^ = (provided fish feed + provided shrimp feed) ÷ (final fish biomass + final shrimp biomass), (10)

Survival^s,f^ (%) = [(final number of animals) ÷ (initial number of animals)] × 100 (11)

^s^ Formula used to assess shrimp growth performance. ^f^ Formula used to assess fish growth performance. ^is^ Formula used to assess the performance of the overall integrated system.

### 2.6. Sludge Production

The volume of sludge produced in each treatment was measured using the values of final and initial TSS and the amount of sludge removed from the units through settling chambers, according to formula (12). To measure the amount of sludge removed using settling chambers, each time they were used a 5 mL sample was taken to measure the TSS and a graded bucket was used to measure the removed volume, then formula (13) was applied. The sludge: biomass relationship was calculated using the total biomass of shrimp and fish in each experimental unit.

SP (kg) = {[(TSS_final_ × V) − (TSS_initial_ × V)] ÷ 1,000,000)} + SR, (12)

where,

TSS_final_ (mg L^−1^) = final concentration of TSS;

V (L) = tank volume;

TSS_initial_ (mg L^−1^) = initial concentration of total suspended solids;

SR (kg) = amount of sludge removed from the system using settling chambers; formula (13).

SR (kg) = (V_sludge_ × W_sample_) ÷ V_sample_, (13)

where,

Vsludge (L) = volume of sludge removed by the settling chamber;

Wsample (kg) = dry weight of the sludge sample in kg;

Vsample (L) = volume of the sludge sample collected.

### 2.7. Water Microbiology Analysis

The counts of total heterotrophic bacteria and *Vibrio* spp. were performed at the beginning and end of the experiment. Samples for the initial counts were taken after the experimental units were filled and before the addition of microalgae and fish feed. For the final counts, samples were taken in each treatment.

Ten mL water samples were collected in 15 mL sterile assay tubes with screw caps. One mL samples were inoculated in serial dilutions using tubes with saline solution (3% NaCl) and 100 µL aliquots were seeded in Petri dishes containing Marine Agar and Thiosulfate-Citrate-Bile salts-Sucrose Agar (TCBS) for viable heterotrophic bacteria and *Vibrio* spp. counts, respectively. Thereafter, they were incubated in a bacteriological stove at 30 °C for 24 h and their counts performed in the following day through colony forming units (CFU mL^−1^) plate counting.

### 2.8. Statistical Analysis

All data were submitted to Bartlett and Shapiro–Wilk tests to evaluate homoscedasticity and normality. Percentage data were arcsine transformed, while microbiology data were log_10_ transformed. Fish FCR was evaluated using a *t*-test. A two-factor ANOVA was used to assess differences between treatments, with fish feed (ff) and microalgae (m) as factors and their interaction expressed as ff × m. Whenever significant differences were found for the interaction, Tukey’s test followed. All tests were performed using a significance level of 5%.

## 3. Results

### 3.1. Water Quality

A significant difference was found only for chlorophyll-*a* concentration between treatments with or without the addition of microalgae (*p* < 0.05) (Table 1). There was no significant interaction between factors.

### 3.2. Growth Performance

#### 3.2.1. Shrimp

There were no significant differences among treatments for shrimp growth performance and no significant interaction (*p* ≥ 0.05) (Table 2).

#### 3.2.2. Fish

No significant interaction between factors were found for fish growth performance (Table 3). Fed fish exhibited significantly higher final mean weight, DGC, and biomass when compared to fish receiving no feed (*p* < 0.05). There was no significant difference in FCR between the treatments with or without microalgae addition (*p* ≥ 0.05).

When comparing the addition of microalgae, fish of those treatments that received it exhibited significantly higher survival and biomass when compared to fish of the treatments that did not receive microalgae addition (*p* < 0.05) (Table 3).

#### 3.2.3. Integrated System

There was no significant interaction between factors for the integrated system performance (Table 4). In the treatments that employed fish feed, the overall FCR was lower when compared to those without the supplementation of fish feed (*p* < 0.05). Biomass and yield were higher in the treatments in which fish were fed when compared to treatments employing no fish feed (*p* < 0.05).

### 3.3. Sludge Production

No significant differences were found for sludge production, sludge: biomass relationship and TSS (*p* ≥ 0.05) (Table 5).

There was a significant interaction between factors for volatile suspended solids (VSS). In the treatments that fish were fed, the VSS percentage was higher when the microalgae was added (*p* < 0.05). As regards the fixed suspended solids, there was a significant difference between the treatments with or without microalgae addition, in which the latter exhibited higher values (*p* < 0.05).

### 3.4. Water Microbiology

Total heterotrophic bacteria and *Vibrio* spp. counts in the beginning of the experiment were 10^5^ and 10^3^ CFU mL^−1^, respectively.

**Figure 2:**
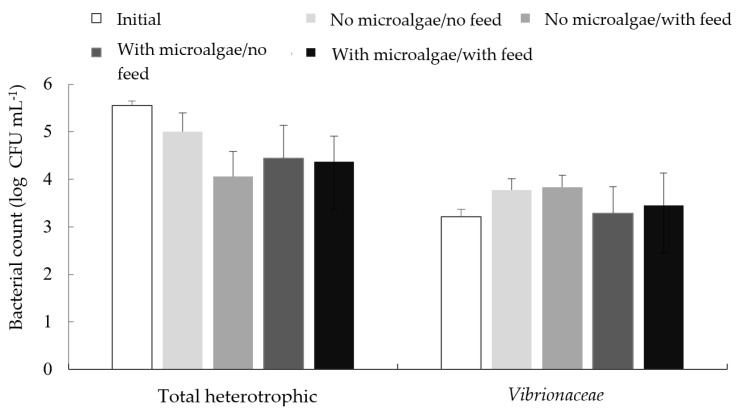
Total heterotrophic bacteria and *Vibrio* spp. counts in the water of a Pacific white shrimp (*Litopenaeus vannamei*) and Nile tilapia (*Oreochromis niloticus*) integrated culture using biofloc technology for 62 days, in which the addition or no addition of both microalgae and fish feed was evaluated in a factorial design.

At the end of the experiment no significant differences among treatments were found for total heterotrophic bacteria and *Vibrio* spp. counts (Figure 2).

## 4. Discussion

### 4.1. Water Quality

In this experiment, the water quality variables exhibited a similar pattern among treatments. Salinity, pH, and alkalinity were maintained at recommended levels for both species and for biofloc development [6,29,36]. The reuse of biofloc water from a matrix tank hastens floc formation and the establishment of nitrifying bacteria [37]. In this work, the mature biofloc inoculum was capable of maintaining safe levels of ammonia and nitrite for the shrimp [29,38] and fish [36,39] throughout the entire experiment as a result of the already developed nitrifying community [14,37,40]. Nitrate and orthophosphate concentrations increased from beginning to end. Still, the nitrate concentration did not reach lethal levels for the species [41,42]. The recurrent addition of microalgae kept high chlorophyll-*a* concentrations in the treatments in which it was added.

Although microalgae can uptake nitrogen and phosphorus [20] their addition to the integrated system did not result in statistically significant differences between treatments for the concentrations of the nitrogenous compounds and orthophosphate. This is in accordance with Lima et al. [43] who evaluated the addition of *Chlorella vulgaris* to the culture of Nile tilapia in biofloc technology and found no significant effects on the concentrations of the aforementioned compounds. Perhaps the concentration of microalgae at the experimental units (5 mg L^−1^ in the present study) was not sufficient to reduce the concentration of these compounds.

### 4.2. Growth Performance

#### 4.2.1. Shrimp

The results obtained in this study indicate that the addition of both microalgae and fish feed had no effect on shrimp growth performance. The mean WGR of 1.1 ± 0.1 g week^−1^ was similar to those reported by other authors for shrimp monoculture using biofloc technology [8,44]. The reason for the lack of differences may be that, because the shrimp were not underfed as the fish, the bioflocs and the microalgae would have been proportionally less important in their nutrition. This idea is supported by Martins et al. [14], in which Pacific white shrimp and Nile tilapia were cultured in a similar integrated system under mature and heterotrophic biofloc systems. Although the authors found that the fish exhibited improved growth performance in the heterotrophic treatment, the shrimp were unaffected, which they argued was due to the latter having been fed according to normal rates as suggested by a feeding table, in contrast to the fish which were underfed [14].

The use of artificial substrates improves shrimp survival when they are cultured under high stocking densities [8,45]. In this study, mean survival was 78.3 ± 7.1%, lower than studies employing similar conditions, likely due to the high stocking density (400 shrimp m^−3^), which indicates that the carrying capacity of the system was reached [5]. Legarda et al. [46] reported a shrimp survival of 91% when the animals were cultured in an integrated system with mullets (*Mugil curema*) using biofloc technology, although the stocking density used was 250 shrimp m^−3^. Similarly, Martins et al. [14] described a shrimp survival of 88% when the animals were reared in integration with *O. niloticus* under the stocking density of 300 shrimp m^−3^. Despite the survival observed in our study being lower when compared to other works, the high stocking density allowed the achievement of higher yields than those reported in other studies [46,47]. For instance, Legarda et al. [46] and Pinheiro et al. [47] used a stocking density of 250 shrimp m^−3^ and found yields of approximately 2.6 kg m^−3^ and 2.1 kg m^−3^, respectively; whereas in the present study yield reached 3.67 kg m^−3^ to 3.88 kg m^−3^ with a stocking density of 400 shrimp m^−3^. This indicates that this shrimp stocking density could be used if the aim is the maximization of yield, considering that, despite the lower shrimp survival, yield still increased when compared to those of other studies. FCR was similar to those obtained in other works for shrimp reared in bioflocs [14,44].

#### 4.2.2. Tilapia

Bioflocs significantly contribute to Nile tilapia nutrition with the efficiency this species exhibits of using bioflocs as a supplemental food source has been described by Avnimelech [48] and Azim and Little [49]. However, floc nutritional quality is greater when they are composed by high VSS percentages, as a result of the greater amount of organic matter relative to mineral matter [14]. In this study, due to the use of a mature biofloc inoculum, the nitrifying bacteria were already established in the system. Consequently, there was no need of adding organic carbon to control ammonia through the heterotrophic assimilation pathway and the VSS percentage remained below 50%. Nonetheless, fish growth performance was similar to those reported for Nile tilapia subjected to feed restriction when cultured in integration with Pacific white shrimp in heterotrophic biofloc systems [13,14]. These results reinforce evidence that previous publications have on the potential of bioflocs being a food source for Nile tilapia [1,48,49]. According to Avnimelech [48], bioflocs can contribute with up to 50% of Nile tilapia protein requirement. In another study, the daily uptake of nitrogen (N) by Nile tilapia reared in biofloc technology was evaluated through ^15^N tracing and the authors found that it was equivalent to 25% of the normal daily protein uptake for this species [1]. In our study, the daily feed supplementation at the rate of 1% of their biomass allowed gains of 58% in final mean weight and 0.56% day^−1^ in DGC when compared with fish that received no feed. Moreover, FCR was lower when compared to works in which fish were fed normal rates and the overall performance was similar to Nile tilapia reared in bioflocs under densities greater than 500 fish m^−3^ [43]. Cavalcante et al. [50] evaluated feeding rate restrictions of 15% and 30% compared to normally fed Nile tilapia and reported a FCR of 1.0 without significant differences between treatments due to the adjustment of feed allowances according to gains in biomass. In the present study, feed restriction was greater than 80% when compared to the normal feeding rates recommended by Ostrensky and Boeger [51]. This could be the reason for the lower FCR observed (0.23 to 0.24), as compared with those found by Cavalcante et al. [50].

Fish survival in this study was similar to that reported in recent studies in which Nile tilapia was cultured using biofloc technology [43,52]. The higher survival in treatments that employed microalgae addition suggests that *S. obliquus* provided nutrients that were not available in the treatments receiving no addition. The nutritional value of bioflocs depends mainly on the carbon: nitrogen ratio and the organic carbon source used [53,54]. Because in this study there was no organic carbon supplementation, biofloc nutritional value was likely lower when compared to heterotrophic systems [14]. Diets containing inadequate levels of amino acids, lipids, and vitamins cause immunosuppression and oxidative stress in fish [55]. Microalgae can produce biocompounds that act as antioxidants and immunostimulants, such as carotenoids and phenolic compounds [56]. Lutein, β-carotene, and vitamins C and E are examples of compounds exhibiting antioxidant and immunostimulant potentials that are produced by the species *S. obliquus* [57,58]. In fact, Nile tilapia cultured in a biofloc system receiving addition of *Chlorella vulgaris* and *S. obliquus* demonstrated improvements in their immune system [19]. Furthermore, Lima [59] demonstrated that *S. obliquus* protein extracts exhibited antioxidant activity in a laboratory setting.

#### 4.2.3. Integrated System

Considering that no significant differences were found for shrimp performance, the significant differences noted in the overall integrated system performance were mainly due to the provision of fish feed and the consequent differences in fish performance. As regards the provision of fish feed, it allowed gains of 8% in biomass and yield as a result of the higher fish biomass achieved, and their values were similar to those reported by other authors studying integrated systems with the same two species. For instance, Poli et al. [52] observed biomass and yield of 3.56 kg and 3.99 kg m^−3^, respectively, whereas Martins et al. [14] found a biomass of 3.81 kg using a heterotrophic-based biofloc system. 

As for the FCR, in the treatments employing fish feed, the higher biomass compensated the higher feed provision compared to the treatments without fish feed supplementation. Thus, even though there was a greater input of feeds in the integrated system, the overall FCR was lower.

### 4.3. Sludge Production

Although there was a greater input of organic matter in the treatments with fish feed, it was possible to increase the overall yield of the integrated system, resulting in similar values of sludge production and sludge: biomass relationship between treatments. The combination of microalgae addition and fish feed provision increased the TSS volatile fraction. However, it was not enough to improve the fish growth performance, as reported by Martins et al. [14] in a heterotrophic biofloc system, in which the fish exhibited an improved growth performance when the percentage of VSS increased. The TSS concentration was kept at suitable values for both species [8,60], through the use of settling chambers.

### 4.4. Water Microbiology

Bacteria and microalgae can interfere positively or negatively in each other’s growth due to the production of biocompounds or competition for nutrients. Bacteria can respond both to living as well as dead cells [61]. Under the conditions of this experiment, namely, microalgae addition under the concentration of 5 mg L^−1^ twice a week, low light penetration and high concentration of organic matter, there were no significant differences in the counts of total heterotrophic bacteria and *Vibrionaceae* at the end of the experiment. Although there are reports that microalgae may be effective in controlling *Vibrio* spp. [23,24], the interaction microalgae-bacteria is very specific and complex [62] and it has already been established that light and nutrient variation affect the interaction of *Scenedesmus acutus* and heterotrophic bacteria [63]. These factors could be responsible for the lack of differences in total heterotrophic bacteria and Vibrionaceae between the two treatments. Still, bacterial counts at the beginning and end of the experiment were similar, with values being comparable to those reported for chemoautotrophic biofloc systems [40].

## 5. Conclusions

The results of this experiment emphasize the potential Nile tilapia exhibits of utilizing bioflocs as a food source. However, they indicate that bioflocs contribute only partially to meet the nutrient requirements of fish. In this sense, the supplementation of fish feed was an important factor that significantly contributed to their growth. The provision of feed at the rate of 1% of fish weight increased their biomass without affecting the sludge production and water microbiology. Additionally, fish feed reduced the FCR and yield of the overall integrated system. The addition of *Scenedesmus obliquus* under the concentration of 5 mg L^−1^ tank^−1^ twice a week increased fish biomass due to an improvement in their survival. However, it did not significantly influence the performance of the overall integrated system, the sludge production, and water microbiology.

Therefore, both factors, fish feed and microalgae addition, can be applied to the integrated rearing of shrimp and tilapia. It is recommended that fish should be fed at the rate of 1% of their biomass when reared in integration with shrimp using biofloc technology, to provide a supplementation of nutrients required for fish growth. Still, future research should focus on evaluating the economic performance of both approaches, fish feed supplementation and microalgae water inoculation, in order to provide further evidence of the feasibility of their application.

## Figures and Tables

**Table 1:** Water quality physico-chemical variables of a Pacific white shrimp (*Litopenaeus vannamei*) and Nile tilapia (*Oreochromis niloticus*) integrated system using biofloc technology which was carried out during 62 days, evaluating the addition of microalgae and fish feed in a factorial design.

Variables	Treatments			
	No Microalgae/No Fish Feed	No Microalgae/with Fish Feed	With Microalgae/No Fish Feed	With Microalgae/with Fish Feed
pH	8.07 ± 0.14	8.02 ± 0.16	8.02 ± 0.15	8.04 ± 0.16
Alkalinity (mg CaCO3 L^−1^)	157.3 ± 30	150.7 ± 31.3	159.1 ± 31	152.7 ± 31.8
Total ammonia-N (mg L^−1^)	0.17 ± 0.13	0.18 ± 0.12	0.16 ± 0.11	0.17 ± 0.14
Nitrite (N-NO2-) (mg L^−1^)	0.61 ± 0.4	0.58 ± 0.38	0.59 ± 0.38	0.62 ± 0.4
Nitrate (N-NO_3_^−^) (mg L^−1^)	19.1 ± 12.3	18.5 ± 10.6	19.1 ± 11.3	19.8 ± 11.8
Salinity (g L^−1^)	15.5 ± 0.8	15.5 ± 1.3	15.5 ± 0.8	15.5 ± 0.8
Ortophosphate (P-PO_4_^3−^) (mg L^−1^)	4.9 ± 3.5	5.1 ± 3.7	5.2 ± 3.7	5.3 ± 3.9
TSS (mg L^−1^)	476 ± 130	473 ± 131	480 ± 129	484 ± 130
Chlorophyll-*a* (µg mL^−1^) *	0.0077 ± 0.0061	0.0076 ± 0.0048	0.0361 ± 0.0146	0.0461 ± 0.0112

Data presented as mean ± standard deviation. * Significant differences when considering only the effect of microalgae addition. Chlorophyll-*a* (µg mL^−1^) of tanks with no microalgae (0.0076 ± 0.001) and tanks with microalgae (0.0443 ± 0.007389). Differences were considered significant when *p* < 0.05. TSS: total suspended solids.

**Table 2:** Pacific White shrimp (*Litopenaeus vannamei*) growth performance when reared in integration with Nile tilapia (*Oreochromis niloticus*) using biofloc technology for 62 days, evaluating the addition of microalgae and fish feed in a factorial design.

Variables	Treatments			
	No Microalgae/No Fish Feed	No Microalgae/with Fish Feed	With Microalgae/No Fish Feed	With Microalgae/with Fish Feed
Final mean weight (g)	12.49 ± 0.81	12.11 ± 0.39	11.88 ± 0.13	11.93± 0.73
WGR (g week ^−1^)	1.16 ± 0.08	1.13 ± 0.05	1.13 ± 0.1	1.11 ± 0.03
DGC (% day^−1^)	1.66 ± 0.07	1.62 ± 0.03	1.60 ± 0.01	1.60 ± 0.06
Survival (%)	73.82 ± 8.37	78.43 ± 4.58	81.64 ± 3.70	79.29 ± 10.33
FCR	2.12 ± 0.17	2.04 ± 0.19	1.98 ± 0.12	2.07 ± 0.23
Biomass (kg)	2.93 ± 0.18	3.04 ± 0.21	3.1 ± 0.14	3.01 ± 0.26
Yield (kg m^−3^)	3.67 ± 0.22	3.8 ± 0.26	3.88 ± 0.18	3.76 ± 0.33

Data presented as mean ± standard deviation. WGR: weekly growth rate. DGC: daily growth coefficient. FCR: feed conversion ratio.

**Table 3:** Nile tilapia (*Oreochromis niloticus*) growth performance when reared in integration with Pacific white shrimp (*Litopenaeus vannamei*) using biofloc technology for 62 days, evaluating the addition of microalgae and fish feed in a factorial design. Partially presented in Vieira et al. [35].

Variables	Fish Feed Addition	Microalgae Addition		Mean	Two-Factor ANOVA		
		No	Yes		ff	m	ff × m
Final mean weight (g)	No	11.06 ± 0.18	11.23 ± 0.51	11.15 ^b^ ± 0.37	*	ns	ns
	Yes	16.49 ± 1.65	17.86 ± 2.65	17.18 ^a^ ± 2.17			
	Mean	13.77 ± 3.09	14.55 ± 3.96				
DGC (% day^−1^)	No	1.69 *±* 0.07	1.79 *±* 0.05	1.74 ± 0.08 ^b^	*	ns	ns
	Yes	2.24 *±* 0.12	2.36 *±* 0.20	2.30 ± 0.17 ^a^			
	Mean	1.97 ± 0.29	2.07 ± 0.32				
Survival (%)	No	81.91 ± 7.86	94.14 ± 1.06	88.03 ± 8.35	ns	*	ns
	Yes	90.42 ± 5.06	93.61 ± 2.45	92.02 ± 4.06			
	Mean	86.17 ^B^ ± 7.62	93.88 ^A^ ± 1.77				
FCR ^1^	No	-	-	-	-	ns	-
	Yes	0.24 ± 0.01	0.23 ± 0.02	-			
	Mean	*-*	*-*				
Biomass (kg)	No	0.42 ± 0.04	0.49 ± 0.02	0.46 ^b^ ± 0.05	*	*	ns
	Yes	0.69 ± 0.04	0.78 ± 0.11	0.74 ^a^ ± 0.09			
	Mean	0.56 ^B^ ± 0.15	0.64 ^A^ ± 0.17				

Data presented as mean ± standard deviation. * Indicates significant differences by two-factor ANOVA (*p* < 0.05), with fish feed (ff) and microalgae (m) as factors and their interaction expressed as ff × m. Values followed by lowercase letters (a or b) in the same column indicate significant difference when considering only the effect of fish feed addition. Values followed by uppercase letters (A or B) in the same row indicate significant difference when considering only the effect of microalgae addition. ^1^ FCR was analyzed using *t*-test. DGC: daily growth coefficient. FCR: feed conversion ratio. ns: non-significant. -: indicates that the mean could not be calculated or that the statistical test could not be performed due to this same reason.

**Table 4:** Yield performance of the overall integrated system of Pacific white shrimp (*Litopenaeus vannamei*) and Nile tilapia (*Oreochromis niloticus*) reared using biofloc technology for 62 days, evaluating the addition of microalgae and fish feed in a factorial design.

Variables	Fish Feed Addition	Microalgae Addition		Mean	Two-Factor ANOVA		
		No	Yes		ff	m	ff × m
FCR	No	1.84 ± 0.08	1.68 ± 0.07	1.76 ^b^ ± 0.11	*	ns	ns
	Yes	1.66 ± 0.09	1.63 ± 0.09	1.65 ^a^ ± 0.09			
	Mean	1.75 ± 0.12	1.66 ± 0.08				
Biomass (kg tank^−1^)	No	3.4 ± 0.1	3.6 ± 0.1	3.5 ^b^ ± 0.20	*	ns	ns
	Yes	3.7 ± 0.2	3.8 ± 0.2	3.8 ^a^ ± 0.20			
	Mean	3.6 ± 0.3	3.7 ± 0.2				
Yield (kg m^−3^)	No	3.8 ± 0.2	4 ± 0.1	3.9 ^b^ ± 0.20	*	ns	ns
	Yes	4.2 ± 0.2	4.3 ± 0.2	4.2 ^a^ ± 0.20			
	Mean	4.0 ± 0.3	4.2 ± 0.2				

Data presented as mean ± standard deviation. * Indicates significant differences by two-factor ANOVA (*p* < 0.05), with fish feed (ff) and microalgae (m) as factors and their interaction expressed as ff × m. Values followed by lowercase letters (a or b) in the same column indicate significant difference when considering only the effect of fish feed addition. FCR: feed conversion ratio. ns: non-significant.

**Table 5:** Sludge production of a Pacific white shrimp (*Litopenaeus vannamei*) and Nile tilapia (*Oreochromis niloticus*) integrated system using biofloc technology which was carried out during 62 days, evaluating the addition of microalgae and fish feed in a factorial design.

Variables	Treatments				Two-Factor ANOVA		
	No Microalgae/No Fish Feed	No Microalgae/with Fish Feed	With Microalgae/No Fish Feed	With Microalgae/with Fish Feed	ff	m	ff × m
Sludge production (kg tank^−1^)	1.01 ± 0.19	1.12 ± 0.05	1.02 ± 0.09	0.98 ± 0.15	ns	ns	ns
Sludge: biomass relationship	0.30 ± 0.05	0.30 ± 0.05	0.28 ± 0.03	0.26 ± 0.05	ns	ns	ns
TSS (mg L^−1^)	476 ± 130	473 ± 131	480 ± 129	484 ± 130	ns	ns	ns
VSS (%)	47.7 ^a^ ± 2.7	47.3 ^a^ ± 2.4	48.1 ^a,b^ ± 2.5	49.0 ^b^ ± 2.4	ns	*	*
FSS (%) ^1^	52.7 ± 4.2	52.9 ± 2.8	51.9 ± 2.5	51.0 ± 2.4	ns	*	ns

Data presented as mean ± standard deviation. * Indicates significant differences by two-factor ANOVA (*p* < 0.05), with fish feed (ff) and microalgae (m) as factors and their interaction expressed as ff × m. ^1^ Significant difference when considering only the effect of microalgae addition. FSS (%) of tanks with no microalgae addition (52.8 ± 3.5) and with microalgae addition (51.5 ± 2.5). Different letters between means indicate significant differences according to Tukey’s test (*p* < 0.05). TSS: total suspended solids. VSS: volatile suspended solids. FSS: fixed suspended solids. ns: non-significant.

## Data Availability

Data supporting the findings of this study can be requested to the corresponding author upon reasonable request.
